# Review on Epidemiology and Public Health Significance of Hydatidosis

**DOI:** 10.1155/2020/8859116

**Published:** 2020-12-03

**Authors:** Abebe Tesfaye Gessese

**Affiliations:** University of Gondar, College of Veterinary Medicine and Animal Sciences, P.O. Box 196, Gondar, Ethiopia

## Abstract

Hydatidosis is a zoonotic parasitic disease caused by larval stages (hydatid cysts) of cestodes belonging to the genus *Echinococcus* and the family *Taeniidae.* Hydatid cyst, which is the larval stage of *Echinococcus,* is a bladder-like cyst formed in various organs and tissues following the growth of the oncospheres of an *Echninoccus* tape worm in that specific organ or tissue. The epidemiology and control of hydatidosis is often considered to be a veterinary matter since the disease can be regulated by controlling parasites in animals. However, collaboration between veterinarians and public health workers is essential for the successful control of hydotidosis. Therefore, the objective of this paper was to review The epidemiology, economic and public health importance of hydatidosis. The developmental stage of *Echinococcus* is that eggs develop to oncospheres, this oncospheres develop to hydatid cyst in the intermediate host and the hydatid cyst if consumed by final host develops to the adult *Echinococcus*. Human echinococcosis is a zoonotic infection caused by the tape worm of the genus *Echinococcus. Echinococcus granucosus granulosis* cause cystic echinococcosis (CE), *Echinococcus multilocularis* cause alveolar echinoloccosis (AE), and *Echinococcus vogeli* and *Echinococcus oligarthus* cause polycystic echinococcosis (PE). From these *Echinococcus mulitilocularis* is rare but is the most virulent, *Echinococcus vegeli* and *Echinococcus oligarthus* are the rarest. Hydatidosis is a zoonotic cosmopolitan parasitic disease found in almost all countries of the world. This disease causes a significant economic loss directly by causing organ or carcass condemnation and indirectly by affecting human and animal health which increase the cost for diagnosis, treatment and control of the disease. Public awareness creation about the transmission and control of the disease and its public health significance and collaboration between veterinarians and public health workers in the prevention and control of the disease is mandatory.

## 1. Introduction

Hydatidosis is a zoonotic parasitic disease caused by larval stages (hydatid cysts) of cestodes belonging to the genus *Echinococcus* and the family *Taeniidae* [1]. Hydatid cyst, which is the larval stage of *Echinococcus,* is a bladder-like cyst formed in various organs and tissues following the growth of the oncospheres of an *Echninoccus* tapeworm in that specific organ or tissue [2]. The larval stage develops in a very wide range of intermediate host including man and the adult stage is found in carnivores [3].

Echinococcosis/Hydatidosis is endemic in sheep and cattle raising areas worldwide. Especially *E. grnulosus* has a worldwide geographical distribution and occurs in all countries. The high prevalence is found in parts of Eurasia, Africa, Australia, and South America. *E. multilocularis* is also distributed in the Northern hemisphere, including endemic regions in central Europe, most of Northern and Central Eurasia, parts of North America and in Northern Africa (Tunisia) [4, 5].

A study on hydatidosis in Ethiopia prevailed prevalence 46% in medium altitude, 24% in high land, and 25% in lowland zones. In a similar study, 14.45% of stray dogs were found to harbor *E. granulous*. These indicate that hydatidosis is one of the major parasitic diseases in Ethiopia [6].

In human beings, hydatidosis is also found worldwide and causes serious public health problems, *E.g. granulosus* is more common and *E. mutilocularis* is the most virulent [7]. From several regions of the world, there are alarming indications of increasing human health risks caused by echinococcosis [8].

In addition to its animal and human health impact, hydatidosis causes economic losses due to organ and carcass condemnation because of the costs for the control and treatment of diseases in animals and humans [7].

The epidemiology and control of hydatidosis are often considered to be a veterinary matter since the disease can be regulated by controlling parasites in animals. However, the collaboration between veterinarians and public health workers is essential for the successful control of hydatidosis [7]. The asymptomatic (subclinical) nature of the disease, both in the final and intermediate hosts, makes the diagnosis difficult and increases the risk of transmission [9, 10]. The control of any infectious agent requires a sound knowledge of the transmission cycles, which perpetuate the agent in nature [1]. Therefore, the objective of this paper was to review the epidemiology, economic and public health importance of hydatidosis.

## 2. Hydatidosis

### 2.1. Etiology

Hydatidosis is caused by the cystic stage of *Echinococcus* species, i.e., hydatid cyst. Hydatid cyst is a large fluid filled cyst lined with germinal epithelium from which invaginated scolices which lie free or in bunches are produced, surrounded by germinal epithelium (brood capsules); the contents of the cysts other than the fluid, i.e., scolices and brood capsules, are frequently described as ‘Hydatid Sand,' occasionally also formed endogenously if the cyst wall ruptures exogenously [3, 10].

Hydatid cyst fluid is pale yellow with 17–200 mg protein per 100 ml cyst fluid has a striking similarity to the serum of the host, and it contains immunoglobulin [11]. The outer covering of the hydatid cyst is formed by connective tissue under which there is the germinal epithelium. Germinal layers are present both in large cyst and daughter cysts, from which the scolices arise [9].

The taxonomy of *Echinococcus* is complex and there appear to be four species, of which some have strains or biotypes that may be recognized on the basis of biochemical, morphological, genetic characteristics, biological behavior, and ecology [12].

*Echinococcus granulosus (E. granulosus)* (Dwarf dog tape worm) is a tapeworm found in the small intestine of definitive hosts and the cystic stage, i.e., hydatid cyst found in various organs (liver and lung) in the intermediate host and occupies a large portion of functional tissues [10, 13]. In sheep, about 70% of hydatid cysts occur in the lungs. In horses and cattle, more than 90% of cysts are usually found in the liver [3]. It is only about 6.0 mm long and consists of a scolex and three or four segments, the terminal gravid one occupying about half the length of the complete tapeworm. Each segment has a single genital opening. The scolex has the rostellum, which is armed with hooks; the ovary is kidney shaped [3, 9].

*Echinococus multilocularis (E. multilocularis)* (Dwarf fox tapeworm) is a tapeworm found in the small intestine of the definitive hosts and the larval stage found mainly in the liver and also in lungs, brain, muscles, lymph nodes, and other organs and tissues [10]. It is a very small tapeworm (2–4 mm) and is generally similar to *E. granulosus*, but usually with 3–5 segments. The terminal segment measures less than half the length of the whole worm. The scolex has four suckers and possesses a double row of large and small hooks. The third segment of the adult tapeworm is sexually mature and the genital pores are in front of the middle of each segment. The uterus is saclike with no lateral sacculations in the terminal proglottid. Gravid segment contains around 200–300 spherical eggs [3].

*Echinococcus vogeli (E. vogeli)* is a tapeworm found in the small intestine of definitive hosts, i.e., bush and domestic dogs. In the intermediate host, the cyst is found in the liver, lungs, and other visceral organs. It is a very small tapeworm (4–6 mm) and usually has 3 segments, the terminal gravid segment being very long in comparison to the rest of the tapeworm. The uterus is saclike, long, and tube shaped. The metacestode has a polycystic structure [10]. It possesses up to 36 large and small rostellar hooks on the scolex, distinguishing it from the other *Echinococcus* species [14].

*Echinococus oligarthus (E. oligarthus)* is a tapeworm found in the small intestine of definitive hosts and viscera, musculature, and skin of intermediate hosts. It is an extremely small tapeworm (2.5–3.0 mm) and usually has 3 segments. The uterus is a sac like in the gravid proglottid [10].

### 2.2. Biology and Epidemiology of Hydatid Cyst/Echinococcus

#### 2.2.1. Life Cycle

The developmental stage of *Echinococcus* is that eggs develop into oncospheres. These oncospheres develop to hydatid cyst in the intermediate host and the hydatid cyst, if consumed by the final host, develops to the adult *Echinococcus* [9]. The prepatent period in the final host is around 40–50 days and its predilection site is the small intestine). The oncospheres of *Echinococcus* are capable of prolonged survival outside the host, being viable on the ground for about two years [10]. The adult *Echinococcus* sheds only one gravid segment per week [3]. The eggs are released from the segment and later swallowed by grazing cattle, sheep, and horses. Infection through drinking water contaminated by windblown eggs is also possible. People become infected through swallowing eggs attached to inadequately washed vegetables, and possibly eggs may be inhaled in dust or carried by flies to uncovered foods [15].

After ingestion by the intermediate host, the oncosphere penetrates the gut wall and circulates in the blood (portal circulation) to the liver or in the lymph to the lungs. These are the two commonest sites for larval development, but occasionally oncospheres escape into the general systematic circulation and develop in other organs and tissues. The oncospheres develop to become hydatid cyst, which is the infective stage. The growth of the hydatid is slow, with maturity being reached in 6–12 months. In the liver and lungs, the cyst may have a diameter of up 20 cm, but in the rarer sites such as the abdominal cavity, where unrestricted growth is possible, it may be very large and contain several liters of fluid. The cyst capsule consists of an outer membrane and inner germinal epithelium from which, when cyst growth is almost complete, brood capsules become detached and exist free in the hydatid fluid. The brood capsule and the scolices collectively are often referred to as “hydatid sand” [9, 10].

Sometimes, complete daughter cysts are formed either inside the mother cyst or externally; in the latter case, they may be carried to other parts of the body to form new hydatids [3]. There are some cysts that do not produce brood capsules or protoscolices, which are called sterile cysts. Cysts in cattle are frequently sterile and pigs, although not commonly infected, usually have sterile cysts. The hydatid cysts in sheep and horses are multilocular and unilocular, respectively. In man, hydatid cysts are found in a wide variety of organs [11]. Little local reaction is shown by most animals to the growing hydatid, which appears as a thin walled cyst, particularly embedded in the organ, but in horses, a thick fibrous capsule develops around the cyst [3].

The final hosts are infected by ingestion of the meat or organ containing the cyst, but when infection occurs in humans, the cycle comes to a dead end because hydatid cysts in humans are unlikely to be eaten by dogs [9, 16].

#### 2.2.2. Host Range

Echinococcus *granulosus* has two biotypes (*E*. *granulosus granulosus* and *E*. granulosus *equines*) that are host adaptive. Dog, red fox, and many wild canids are the common definitive hosts. The intermediate stage of *E. g. granulosus* is found in domestic ruminants, man, pigs, and wild ruminants, whereas horse and donkey are resistant. The larval stage of *E. granulosus equines* is found in horses and donkeys but not in humans [3].

Foxes serve as the principal definitive hosts for the adult of *E. multilocularis*, but dogs, cats, and coyotes can also serve that function. Larval forms occur in various rodents, chiefly voles, field mice, shrews, and ground squirrels. Humans can also be infected [17].

*Echinococcus vogeli* is a parasite of the bush dog and occasionally domestic dogs, with an intermediate stage in pacas and other rodents, and on occasion, humans [17].

Wild felids like the cougar, jaguar, and cerot are important definitive hosts of *Echinococcus oligarthus* and the larval stage found in agoutis, rodents, spiny rat, paca, and man can be an accidental host [10].

#### 2.2.3. Geographical Distribution

Hydatidosis is endemic in sheep and cattle raising areas worldwide. However, its prevalence is very high in the Mediterranean region [5].

*Echinococcus grannulosus* has a worldwide geographic distribution and occurs in all countries. High parasite prevalence is found in the Mediterranean region, Russian Federation, and adjacent independent states, Republic of China, Africa (Northern and Eastern Regions), Australia and South America [8]. The synanthropic cycle with domestic dogs as final hosts and sheep or other livestock animals as intermediate hosts predominates as an infection source for humans worldwide [8].

The epidemiology of *Echinococus granulosus granulosus* is based on two cycles, pastoral and sylvatic. In the pastoral cycle, the dog is always involved, being infected by feeding of ruminant offal containing hydatid cysts. The domestic intermediate host will vary according to the local husbandry, but the most important is the sheep, which appears to be the natural intermediate host, scolices from these animals being the most highly infective for dogs. The pastoral cycle is the primary source of hydatidosis in man, infection being by accidental ingestion of oncospheres from the coats of dogs, vegetables, and other food stuffs contaminated by dog feces [3]. The sylvatic cycle occurs in wild canids and ruminants and is based on predation or carrion feeding. It is less important as a source of human infection except in hunting communities where the infection may be introduced to domestic dogs by the feeding of viscera of wild ruminants (Table 1).

Equine hydatidosis caused by *Echinococcus granulosus equines* is commonest in Europe, and in other parts of the world, most cases have been recorded in imported European horses. The strain is highly specific for the horse, and the eggs do not develop in sheep. The domestic dog and the red fox are the final hosts, and the cycle in countries of high prevalence depends on access by dogs to infected equine viscera. In Europe, the most likely source is offal from horse abattoirs. The horse strain does not appear to be infective to man [3, 10].

The natural life cycle of *E. multilocularis* is based upon the predator-prey relationship that exists between carnivores and small mammals [4]). The sylvatic cycle of *E. multilocularis* is restricted to wild animal hosts, predominantly to foxes as definitive hosts and small mammals, mainly rodents as intermediate hosts. *E. multilocularis* eggs excreted by definitive hosts to the environment are ingested by intermediate hosts in which the metacestode stage with protoscolices develops. Infected intermediate hosts are the prey of wild carnivores. Evidence suggests that the sylvatic cycle of *E. multilocularis* is the predominant source of infection for humans and for other aberrant hosts in most of the endemic regions. In Japan, during 1905–1991, the average prevalence of *E. multiloculari*s was 14% in 58,073 foxes and only 1% in 9,742 dogs [8]).

**Figure 1:**
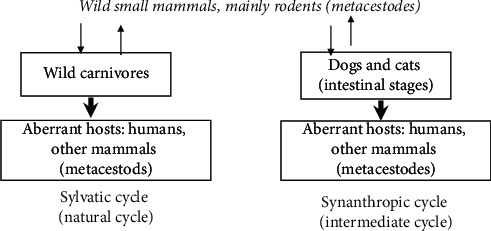
Epidemiologically relevant cycles of *E. multilocularis* (Source: [4]).

Though *E. multilocularis* has a wide distribution in the northern hemisphere, including North America, Green land, Scandinavia, central Europe, Russia, Middle East, India, China, and Japan, it is essentially a parasite of tundra regions with its greatest prevalence in the subarctic regions of Canada, Alaska, and Russia. Its basic epidemiological cycle in these regions is in the arctic fox and wolf and their prey, small rodents and insectivores. In North America, its range is extending from Canada to the United States, where the red fox and coyote act as final hosts. The cycle is, therefore, sylvatic [3] (Figure 1).

*Echinococcus vogeli* and *Echinococcus oligarthus* occur in Central and South America; other details for both species are similar to those for *E. multilocularis* [10]. In human beings, they cause polycystic echinococcosis (PE) and distributed in central and South America [8]).

**Figure 2:**
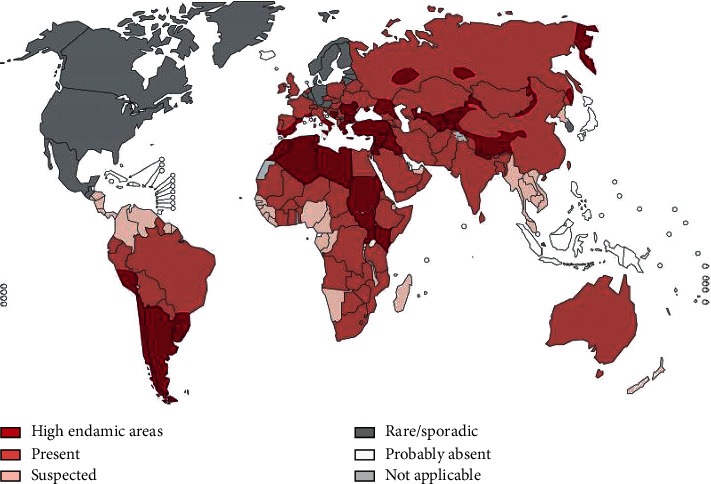
Distribution of *Echinococcus granulosus* and cystic echinococcosis worldwide (Source: [18].

Cystic echinococcosis is globally distributed in most pastoral and rangeland areas of the world, with highly endemic areas in the eastern part of the Mediterranean region, northern Africa, southern and eastern Europe, at the southern tip of South America, in Central Asia, Siberia, and western China (Figure 2, [18]).

### 2.3. Pathogenesis and Clinical Signs

In the intermediate hosts, clinical signs depend on the organs involved (location of the cyst) and level of infection, but usually, no visible clinical symptoms are observed [13]. The hydatid in the liver or lungs is usually tolerated without any clinical signs, and the majority of infections are only revealed at the abattoir. Where oncospheres have been carried in the circulation to other sites, such as the kidney, pancreas, central nervous system, or marrow cavity of long bones, pressure by the growing cyst may cause a variety of clinical signs. However, cysts in the liver can cause hepatic insufficiency, digestive disturbances, and ascites, while the cysts in the lungs produce dyspnea. Cysts in the brain produce cerebral symptoms (paralysis, blindness, etc.). When a man is involved as an intermediate host, the hydatid in its pulmonary or hepatic sites results in respiratory distress or abdominal enlargement, respectively, depending on whether the lungs or liver are infected [10, 19].

Diseases mainly result from pressure effects caused by the enlarging cysts (obstruction) and due to hypersensitivity to *Echinococcus* antigen mainly in human beings [16]. Hypersensitivity or anaphylactic reactions to worm antigens occurs if the cyst ruptures [20]. In the liver, invasion by the metacestode stage can result in atrophy of the parenchyma and cause cirrhosis. Expansion of alveolar echinococcosis in the liver produces aggregates of small gelatinous cysts that appear similar to malignant neoplasia [10].

Infection in the definitive hosts is subclinical, usually symptomatic [10, 21]. The adult tapeworm is not pathogenic, and thousands may be present in a dog without clinical signs [3].

### 2.4. Diagnosis

Diagnosis of infection in a dog with an adult tapeworm is difficult because the segments are small and are shed sparsely; however, the presence of the adult worm in the intestinal tract can be verified by the demonstration of segments and/or ova of *Ehinococus* in the feces. Identification of the egg is based on their size (2.0–3.0 mm), shape which is ovoid. The eggs have a thick shell with radial striation (embryophore). The six hooks of the hexacanth embryo allow it to be distinguished from pollen grains or other debris [17, 21].

The whole adult *Echinococcus* can also be demonstrated in the feces of definitive hosts (mostly dogs) purged with praziquantel or other anti \helmentics [14]. Morphologically, the worms are very small. Only a few number of segments are present, usually three, with the terminal gravid, the middle mature, and the anterior immature. The scolex has the rostellum which is armed with hooks. The ovary is usually kidney shaped specially in *E. granulosus* [9]. If necropsy is available, the small intestine should be opened and immersed in shallow water, when the attached tapeworms will be seen as small, slender papillae [10]. The sedimentation and counting technique at necropsy is the well-established method for the detection of intestinal *E. multilocularis* in the definitive host, although the intestinal scraping technique is also useful [10].

More recent research techniques include the detection of copro DNA by Deoxyribonucleic acid, PCR (Polymerase chain reaction) and the detection of *E. multilocularis* specific copro antigen in an ELISA (Enzyme linked immunosorbent assay) based assay are practiced [10].

In the intermediate hosts, the presence of hydatids as a clinical entity is rarely suspected and specific diagnosis is never called for [3]. Even most cases are observed and confirmed during postmortem. Diagnosis is with imaging tests, examination of the cyst fluid, or serologic tests (immunodiagnostic tests) [22].

Radio graphic diagnosis can be done as per the location of the suspected area; ultrasound examination, computerized tomography (CT), and magnetic resonance imaging (MRI) techniques may be pathognomonic if daughter cysts and hydatids and are present, but not in simple benign cysts, abscesses, or benign or malignant tumors [22, 23].

A scientist Casoni [24] performed the test which is popularly called cason's test; hydatid cyst fluid is inoculated to the suspected individual and there would be hypersensitivity reaction within 15 minutes or less in positive cases [9].

According to Mandal [9], immunodiagnosis of hydatidosis is also possible and the source of antigen is protoscolices, cyst fluid, and cyst membrane. The fertile cysts contain much amount of antigen. The immnodiagnostic tests are complement fixation test (CFT), indirect fluorescence antibody test (IFAT), indirect halmagglutination test, leukocyte migration inhibition test (LMIT), latex agglutination test, arc 5 double diffusion test, immunoelectrophoresis and radioimmune assay. In addition, there are also other recent trends of diagnosis which includes peroxidase micro-ELISA (Enzyme Linked Immuno Sorbant Assay), Avidin-borin-ELISA (Ab-ELISA) which is used in heparin binding lipoprotein (HBLP) for bovine fertile hydotid cyst.

In man, the methods most commonly used are serologic tests such as complement fixation or immunoelectrophoresis; scanning techniques and cason's test are also used [3].

### 2.5. Treatment

There is no specific treatment of hydatid cysts in domestic animal [13]. *Echinococcus* tapeworms are difficult to treat, but several drugs, notably praziquantel, are now available which are highly effective. After treatment, it is advisable to confine dogs for 48 hours to facilitate the collection and disposal of infected feces [3]. Dogs and cats can be treated with praziquantel or epsiprantel, but treatment of domestic intermediate hosts is not advised [10].

In man, surgical removal offers the best mode of treatment where the cysts are accessible, but recurrence after surgery is common [16]. Mebendazole, Albendazole, and Prazionlantel therapies have been reported to be effective [10]. For *E. granulosus*, albendazole 400 mg orally bid for 1 to 6 months (7.5 mg/kg) is curative in 30%–90% of patients and can be used to suppress growth in inoperable cases. Albendazole is often given before surgery to prevent metastatic infections if there is spillage of cyst contents [22].

The prognosis for *E. multilocularis* infection is poor unless the entire larval mass can be removed. Surgery is indicated if it is feasible, which depends on the size, location, and manifestations of the lesion. Albendazole in the above dose can suppress the growth of inoperable lesions. Liver transplantation has been life-saving in a few patients [16, 22].

### 2.6. Control

*Echinococcosis* can be controlled through preventive measures that break the cycle between the definitive and the intermediate host. These measures include deworming dogs, inspecting meat, and educating the public on the risk of the cyst to humans and on avoiding feeding offals to dogs, as well as introducing legislation. None of these measures will work in isolation; however, the disease can be controlled successfully through health education and appropriate legislation, only when people understand the life cycle of the parasite [7].

Deworming of dogs is based on the regular treatment of dogs to eliminate the adult tapeworm and on the prevention of infection in dogs by exclusion from their diet of animal material containing hydatids. This is achieved by denying dogs access to abattoirs, and where possible, by proper disposal of carcasses. In some countries, those measures have been supported by legislation, with penalties when they are disregard. Proper disposal of the carcass is by deep burial or incineration [10].

The control of stray dogs is an essential means of control. In countries where no specific measures for hydatid control exist, it has been found that an incidental benefit from the distribution of stray dogs for rabies control has been a great reduction in the incidence of hydatid infection [10, 14].

In some countries, control regimes have involved the administration of purgative antihelmentics, for dogs such as arecoline hydrochloride, so that the whole tapeworm is expelled out [3]. Efficient meat inspection procedures with efficient control of rejected meat and offal are appropriate in hydatid control [14]. Transmission to humans can be controlled by eating vegetables by washing properly, keeping foods closed, personal hygiene, and avoiding kissing dogs, preventing the egg from being transferred to humans [16]. A recombinant DNA vaccine has been developed for *E. granulosus,* but it requires further refinement for practical application, and it is currently not available commercially [10].

Control of hydatidosis is less effective without the support of dog owners, and this support can only be obtained if the people have a clear understanding of the life cycle of the hydatid parasite(s) and what risk factors contribute to human infections. Dissemination of this information is the biggest challenge for hydatid control. Participatory planning between dog owners and community leaders should evaluate the possible control technologies and should enable a choice of those aspects that suit the sociology and economic status of the particular community [25].

Prevention of cystic echinococcosis measures also includes restricting home slaughter of sheep and other livestock, not consuming any food or water that may have been contaminated by fecal matter from dogs, washing hands with soap and warm water after handling dogs and before handling food, and teaching children the importance of washing hands to prevent infection [26].

The control of the parasitic zoonoses is encompassed by a complex group of actions because of its existence in the life cycle of animal hosts (domestic and wild) as well as humans. This complexity is the reason why, despite knowing the life cycle of Echinococcus granulosus and their mechanisms of transmission thoroughly, hydatidosis continues to be an important zoonosis in many regions of the world [27].

### 2.7. Public Health Significance

Human *Echinococcosis* is a zoonotic infection caused by the tapeworm of the genus *Echinococcus. Echinococcus granucosus granulosis* causes cystic echinococcosis (CE), *Echinococcus multilocularis* causes alveolar echinococcosis (AE), and *Echinococcus vogeli* and *Echinococcus oligarthus* cause polycystic echinococcosis (PE). From these, *Echinococcus mulitilocularis* is rare but is the most virulent; *Echinococcus vegeli* and *Echinococcus oligarthus* are the rarest [5].

Hydatid cysts can cause serious problems for humans [13]. The adult tape warm occurs in domestic dogs and cats, are potential carriers of the infection for man, which brings it into the veterinary sphere of interest [3]. Human infection follows ingestion of the eggs passed by infected dogs. This may occur by eating raw vegetables or other food items contaminated with dog feces. Fingers contaminated with the eggs and kissing dogs may cause the eggs to be transferred directly to the mouth [16]. On the other hand, the sylvatic cycle of *Echinococcus*, which occurs in wild canids and ruminants, is based on predation or carrion feeding. However, it is less important as a source of human infection but in hunting communities [3].

Prevalence rates are determined by epizootiological factors related to the size of the stray dog population and its worm burden and to the infection rates in the intermediate host reservoir livestock population. Socioeconomic development and sociocultural practices are considered important determinants in the continued transmission of the disease [28].

Infection of human echinococcosis is often acquired during childhood when intimate contact with a pet dog is more likely. But the clinical disease develops only several years later when the hydatid cyst has grown big enough to cause obstructive symptoms. The disease results mainly from pressure effects caused by the enlarging cysts. A second pathogenic mechanism in hydatid disease is hypersensitivity to the *Echinococcal* antigen [16]. But the disease may be diagnosed in a shorter time when the cyst is found in the brain [5]. The rupture of the hydatid cyst causes an allergic (Type I) reaction. Slow leakage of worm antigens ensures that the patient's most cells are sensitized with specific immunoglobulin E (IgE), and the massive flood of antigens on rupture may cause acute fatal anaphylaxis, with vascular collapse and pulmonary edema. Why allergic reactions are such a feature of worm infection is not really clear, but that may be due to some feature of the antigens; in addition, it has been suggested that IgE plays a role in protection against worms [23].

The presence of a large number of stray dogs was an important factor in the spread of CE. They were rarely vaccinated, had easy access to infected offal at slaughtering sites and had insufficient or inappropriate anthelmintic treatment [29].

The endemic areas are the Mediterranean countries, the Middle East, the Southern parts of South America, Iceland, Australia, Newzealand, and Southern parts of Africa. The incidence of CE in endemic areas ranges from 1–220 cases per 100,000 inhabitants, while the incidence of AE ranges from 0.03–1.2 cases per 100,000 inhabitants, making it a much more rare form of echinococcosis [5].

Morbidity is usually secondary to free rupture of Echinococcal cyst (with or without anaphylaxis), infection of the cyst or dysfunction of the affected organ. The source of infection may be local to the lesion or another body site but is usually undiagnosed [23]. Examples of dysfunction of the affected organ are biliary obstruction, cirrhosis, bronchial obstruction, renal outflow obstruction, increased intracranial pressure secondary to hydrocephalus which is due to cerebral outflow obstruction [5].

In CE, mortality is secondary to anaphylaxis, systematic complication of the cyst, cirrhosis, respiratory failure, or operative complication. CE is a disease of younger adults with an average age at diagnosis of 30–40 years. The degree of symptoms is affected by the parasite load, the site, and the size of the cysts [7]. Theoretically, echinococcosis can involve any organ. The liver is the most common organ involved, followed by the lungs. These two organs account for 90% of cases of echinococcosis. For example, the organs affected by *E. granulosus granulosus* are the liver (63%), lungs (25%), muscles (5%), bones (3%), kidneys (2%), brain (1%), and spleen (1%) [5].

In the liver, the pressure effect of the cyst can produce symptoms of obstructive jaundice, abdominal pain; rupture into the bile duct, abdominal, or peritoneal cavity may produce fever, urticaria, and orserious anaphylactic reaction. Involvement of the lungs produces chronic cough, dyspnea, pleuritic chest pain, and hemoptysis [22]. If the cyst ruptures, there is a risk of death from anaphylaxis, or if the person survives, released daughter cysts may resume development in other regions of the body [3]. Headache, dizziness, and a decreased level of consciousness may signify cerebral involvement. Specific neurologic deficits may occur depending on the location of the cyst in the brain. Extreme pain with or without neurologic deficit is a sign of either bone or muscle involvement [5].

In AE, the liver is the primary site of infection, and it closely mimics cirrhosis or carcinoma. Distant metastasis is possible. AF is a disease of older adults with an average age of diagnosis of older than 50 years. In clinical cases of AE, the mortality rate is 50–60%. This figure reaches 100% for untreated or poorly treated AE. Sudden death has been reported with AE in symptomatic patients (autopsy diagnosis) [7].

The expansion of the synanthropic cycle, involving domestic dogs that prey on metacestode infected rodents, may lead to an increase in the prevalence of human AE. *E.multilocularis* egg contamination has been predicted to be maximal where the urban and rural habitats overlap [10].

These facts should be reasons for health authorities to establish internationally coordinated systems of surveillance and risk assessment to improve and support measures for control and prevention [8]).

Cystic echinococcosis has been recorded in 21 out of China's 31 provinces, autonomous regions and municipalities (approximately 87% of the territory). It constitutes one of the major public health problems, especially in several northwestern provinces and autonomous regions [30].

## 3. Conclusions and Recommendations

Hydatidosis is a zoonotic cosmopolitan parasitic disease found in almost all countries of the world, including Ethiopia. This disease causes a significant economic loss directly by causing organ or carcass condemnation and indirectly by affecting human and animal health, which increases the cost for diagnosis, treatment, and control of the disease. Improper disposal of the carcass (organ), increased population of stray dogs, and lack of appropriate legislation for the control of the disease are the most important factors that increase the transmission of the disease. Based on the above conclusive points, the following recommendations are forwarded [29, 31]:

- Regular deworming of pet dogs and control of stray dogs
- Public awareness creation about the transmission and control of the disease and its public health significance
- Proper disposal of carcass either by burning or burring and avoiding the habit of giving offal to dogs
- Collaboration between veterinarians and public health workers in the prevention and control of the disease is mandatory
- Proper food hygiene and personal hygiene especially, those having close contact with pets

## Figures and Tables

**Table 1:** Summary of the characteristics of *Echinococcus* Species.

Character	*Echinococcus granulosus*	*Echinococcus multilocularis*	*Echinococcus vogeli*	*Echinococcus oligarthus*
Geographic distribution	Cosmopolitan	Central and North Eurasia, Northern North America	Central and South America	Central and South America

Definitive hosts	Primarily dogs and other canids	Primarily foxes, also other canids and cats	Wild felids	Bush dog

Intermediate and aberrant hosts	Primarily ungulates also marsupials and primates, humans	Primarily arvicola rodents, also other small mammals, humans	Rodents, agoutis, paca, spiny rats, humans	Primarily agoutis, also other rodents, humans

Nature of cyst	Unilocular, endogenous proliferation, no in filtration or metastasis	Multivesicular, endogenous proliferation, metastasis	Polycystic, exogenous and endogenous proliferation, no metastasis	Polycystic, endogenous and exogenous proliferation, no infiltration or metastasis

Location of cyst	Visceral, primarily liver and lungs	Visceral, primarily liver	Peripheral, primarily muscle	Visceral, primarily liver

Source. [1].
